# When Silence Signals Safety: Governance and Responsibility in AI-Enabled Prescription Verification

**DOI:** 10.7759/cureus.103272

**Published:** 2026-02-09

**Authors:** Esteban Zavaleta-Monestel, Jeaustin Mora-Jiménez, Sebastián Arguedas-Chacón

**Affiliations:** 1 Health Research Department, Hospital Clinica Biblica, San Jose, CRI; 2 Health Research Department, Hospital Clínica Bíblica, San José, CRI

**Keywords:** artificial intelligence governance, automation bias, clinical decision support, medication safety, prescription verification

## Abstract

Artificial intelligence (AI) is increasingly utilized to enhance prescription verification by screening medication orders, prioritizing pharmacist review, and, in certain implementations, suppressing or deprioritizing alerts deemed low risk. While these systems may improve efficiency and the detection of prescribing risks, they also introduce challenges related to clinician reliance, accountability, and system oversight. This editorial argues that AI-enabled prescription verification may shift, in settings where algorithmic triage or alert suppression is relied upon, safety from an active clinical judgment to a passive inference based on algorithmic silence, redistributing rather than eliminating medication safety risk. As a result, safety work transitions from preventing individual errors to maintaining vigilance through continuous monitoring and governance. Key issues discussed include automation bias, data drift and dataset shift, distributed clinical responsibility, and the limitations of traditional validation approaches, such as one-time pre-implementation testing, reliance on static performance metrics, and periodic audits. Addressing these challenges requires governance frameworks that clarify accountability, uphold human judgment, and support ongoing evaluation of AI systems in clinical practice. By framing prescription verification as a socio-technical activity rather than solely a technical function, this editorial advances the discourse on the responsible integration of AI into medication safety workflows.

## Editorial

Artificial intelligence (AI) is increasingly integrated into prescription verification workflows in both inpatient and outpatient settings. Machine learning systems now screen medication orders, prioritize pharmacist review, and, in certain implementations, suppress or deprioritize alerts deemed low risk. These tools are typically introduced as incremental enhancements to existing clinical decision support, with the promise of increased efficiency while maintaining or improving safety. Early implementations indicate that machine learning models may identify prescriptions with a higher likelihood of medication error while reducing unnecessary interruptions in pharmacist workflow [1].

However, these systems also alter the way safety is inferred. In implementations that rely on triage or alert suppression, when a medication order proceeds without algorithmic interruption, the absence of an alert or signal may be interpreted as confirmation of correctness. As adoption expands, prescription verification may shift, in specific settings, from an active, judgment-driven checkpoint to a process increasingly mediated by algorithmic reassurance.

Historically, prescription verification has served as a cognitive safeguard within the medication-use process. Beyond identifying explicit rule violations, it has required clinicians to interpret orders in relation to patient-specific factors such as comorbidities, care trajectories, and clinical intent. AI-enabled verification changes this function. When an order is cleared silently by an algorithm, reassurance is conveyed implicitly rather than through explicit recommendation. Over time, this algorithmic “silence,” defined here as the absence of interruption or alert in triage-based systems, may supplant clinical validation, altering how clinicians determine prescription safety.

This shift exemplifies a well-documented human response to automation. When systems appear selective and reliable, clinicians tend to trust them more and question them less frequently. In medication-related decision support, this phenomenon, known as automation bias, has been shown to influence pharmacist oversight and clinical decision-making, leading clinicians to defer to computerized outputs even when those outputs may be incomplete or incorrect [2]. In prescription verification, automation bias is particularly consequential because confidence is reinforced not by what systems communicate, but by what they omit. The routine clearance of most orders without interruption may normalize silence as evidence of safety.

AI-enabled verification systems also introduce risks that are not immediately apparent at the point of care. Machine learning models depend on data distributions that evolve over time as prescribing practices, formularies, patient populations, and documentation patterns change. Consequently, system performance may degrade gradually without clear indications of failure. Evidence from deployed clinical AI systems demonstrates that data drift and related forms of dataset shift are common sources of performance degradation and that such changes may remain undetected without deliberate monitoring strategies [3]. Additional evidence shows that clinician shift and context-of-use changes can meaningfully alter real-world behavior and system performance, reinforcing the need for ongoing surveillance rather than one-time validation [4].

Although much of this evidence comes from diagnostic and risk-prediction systems, the underlying mechanisms, including data drift, evolving context of use, and changing clinician behavior, are likely to operate similarly in AI-enabled prescription verification systems, albeit with variation depending on clinical setting and implementation maturity. Ongoing clearance of most prescriptions may therefore reinforce clinician trust even as sensitivity to emerging risks diminishes.

When harm eventually becomes apparent, it rarely results from a single missed check or identifiable moment of failure. Instead, it emerges as a diffuse, systemic pattern that is difficult to trace. This challenges traditional approaches to medication safety, which often focus on discrete adverse events rather than the gradual accumulation of vulnerability within complex systems. In this context, safety risk is redistributed rather than eliminated, shifting from frontline error prevention to downstream detection and response.

This redistribution reflects a deeper governance problem. AI-enabled prescription verification tools are frequently treated as technical infrastructure rather than as sources of clinical risk. Responsibility for their design, updating, and maintenance may be distributed across vendors, information technology teams, and operational leadership, while medication safety programs remain accountable for outcomes. This separation creates a structural blind spot in which those held responsible for safety may lack the authority, visibility, or control required to meaningfully oversee how these systems evolve in practice. International guidance on the ethics and governance of AI in health has emphasized that accountability gaps of this kind pose substantial risks if left unaddressed [5].

Traditional safety controls offer limited protection in this environment. One-time pre-implementation validation assumes system stability, reliance on static performance metrics limits sensitivity to change, and periodic audits assume detectability of failure and predictability of system behavior. AI-enabled systems challenge each of these assumptions. Their most significant risks arise not from isolated malfunction, but from interactions among algorithms, workflows, time pressures, and clinician trust. Socio-technical analyses of health information technology have long recognized that safety emerges from the interaction among people, processes, and technologies, rather than from any single component alone [6].

These dynamics also complicate how safety is defined and measured. Reductions in alert volume, improvements in workflow efficiency, and gains in predictive performance are often interpreted as indicators of safer care, yet they primarily describe system behavior rather than clinical safety. Systematic reviews of medication safety-related clinical decision support systems have shown that improvements in alert metrics and process measures frequently fail to translate into reliable harm reduction or improved patient outcomes, particularly when workflow integration and context-of-use considerations are insufficiently addressed [7].

Addressing these challenges requires reframing prescription verification as a socio-technical activity rather than a purely technical function. Governance frameworks must clarify who is responsible for monitoring AI system behavior, how performance changes are detected, and when intervention is required. Effective governance should include designation of an accountable clinical or organizational owner, explicit performance and drift thresholds, and predefined criteria for intervention or model retraining. Medication safety leaders must have visibility into, and authority over, AI-enabled tools that influence prescribing decisions. Emerging regulatory approaches, including guidance from the U.S. Food and Drug Administration on predetermined change control plans for AI-enabled medical devices, increasingly emphasize the need for continuous oversight of adaptive systems rather than reliance on static approval processes [8].

Equally important is the preservation of human judgment. AI should not be framed as a safeguard that replaces clinical reasoning, but as a tool that reshapes it. Verification must remain an active cognitive process, even when systems offer reassurance. When harm arises from workflows algorithmically approved, clinicians may experience erosion of verification skills, moral distress, and ambiguity about accountability. These professional risks deserve explicit recognition alongside technical considerations. Silence should not be equated with correctness, and efficiency should not be mistaken for safety.

AI does not simplify prescription verification; it deepens the process. It shifts the work of safety from enforcing rules to sustaining vigilance, and from preventing known errors to anticipating emerging risks. This editorial is conceptual in nature and does not present original empirical data or evaluate specific commercial systems. Rather, it aims to advance discussion about responsible implementation. Ultimately, realizing the benefits of AI-enabled prescription verification will depend on governance structures that balance efficiency with accountability and automation with sustained clinical judgment. When silence is no longer assumed to signal safety, AI can more effectively support resilient medication-use systems.
